# Contiguous mutation syndrome in the era of high-throughput sequencing

**DOI:** 10.1002/mgg3.134

**Published:** 2015-03-18

**Authors:** Maéva Langouët, Karine Siquier-Pernet, Sylvia Sanquer, Christine Bole-Feysot, Patrick Nitschke, Nathalie Boddaert, Arnold Munnich, Grazia M S Mancini, Robert Barouki, Jeanne Amiel, Laurence Colleaux

**Affiliations:** 1 INSERM UMR 1163, Laboratory of Molecular and Pathophysiological Bases of Cognitive Disorders, Paris Descartes – Sorbonne Paris Cité University, Imagine Institute, Necker-Enfants Malades Hospital75015, Paris, France; 2 Metabolic and Proteomic Biochemistry Service, Necker-Enfants Malades Hospital, AP-HP75015, Paris, France; 3 Genomic Platform, INSERM UMR 1163, Paris Descartes – Sorbonne Paris Cité University, Imagine Institute75015, Paris, France; 4 Bioinformatic Platform, INSERM UMR 1163, Paris Descartes – Sorbonne Paris Cité University, Imagine Institute75015, Paris, France; 5 Service de Radiologie Pédiatrique, Hôpital Necker-Enfants Malades, AP-HP75015, Paris, France; 6 Department of Clinical Genetics, Erasmus University Medical Center3015 CN, Rotterdam, The Netherlands; 7 Service de Génétique, Hôpital Necker-Enfants Malades, AP-HP75015, Paris, France

**Keywords:** AP4 deficiency syndrome, intellectual deficiency, obesity, whole-exome sequencing, zinc-*α*2-glycoprotein

## Abstract

We investigated two siblings, born to consanguineous parents, with neurological features reminiscent of adaptor protein complex 4 (AP4) deficiency, an autosomal recessive neurodevelopmental disorder characterized by neonatal hypotonia that progresses to hypertonia and spasticity, severe intellectual disability speech delay, microcephaly, and growth retardation. Yet, both children also presented with early onset obesity. Whole-exome sequencing identified two homozygous substitutions in two genes 170 kb apart on 7q22.1: a c.1137+1G>T splice mutation in *AP4M1* previously described in a familial case of AP4-deficiency syndrome and the *AZGP1* c.595A>T missense variant. Haplotyping analysis indicated a founder effect of the *AP4M1* mutation, whereas the *AZGP1* mutation arose more recently in our family. *AZGP1* encodes an adipokine that stimulate lipolysis in adipocytes and regulates body weight in mice. We propose that the siblings' phenotype results from the combined effects of mutations in both *AP4M1* and *AZGP1* that account for the neurological signs and the morbid obesity of early onset, respectively. Contiguous gene syndromes are the consequence of loss of two or more adjacent genes sensible to gene dosage and the phenotype reflects a combination of endophenotypes. We propose to broaden this concept to phenotypes resulting from independent mutations in two genetically linked genes causing a contiguous mutation syndrome.

Recent developments in next-generation sequencing (NGS) and whole-exome sequencing (WES) have considerably empowered our ability to identify the genetic basis of monogenic Mendelian traits. Yet, human phenotypes are the result of variants at multiple loci and studies of human diseases must often expand beyond single-locus analyses. Accordingly, the power of NGS has also fostered the identification of families with pathogenic mutations in more than one disease gene. Several examples illustrated the role for digenic inheritance in deafness, long QT, or nephrotic syndromes (Schaffer 2013). Similarly, recent publications illustrate how WES can help identifying genetic modifier of a Mendelian trait (Lemmers et al. 2012; Prokudin et al. 2014).

Homozygous or compound heterozygous mutations in any of the four genes encoding the subunits of the adaptor protein complex 4 (AP4) have been found to cause neurological disease underscoring the existence of a recognizable AP4 deficiency syndrome. The phenotype is characterized by microcephaly, severe intellectual disability (ID) with delayed or absent speech, progressive spasticity leading to wheelchair dependence in early adolescence, and growth retardation. We report here on two siblings (one female and one male) presenting the association of typical AP4-deficiency neurological presentation and severe obesity with early onset and provide evidence that this condition is a contiguous mutation syndrome.

Patients II.1 and II.2 (Table1 and Fig.1B–C) are brother and sister born to first cousin parents originating from Algeria. They were term-born after an uneventful pregnancy and delivery with normal birth parameters. Neonatal hypotonia was reported for both patients. At their last visit at 4 (II.2) and 7 (II.1) years of age, II.1 had a small head circumference (−1.5 standard deviation [SD]) and II.2 was microcephalic with occipitofrontal circumference on −2 SD (Table1). They had severe intellectual deficiency with only a few single words. Eye contact was sustained and they both had purposeful hand skills. Patient II.2 presented unmotivated laughs and episodes of heteroaggressivity. Patient II.1 could walk with wall support from 15 months of age and then lost the ability to walk at 4 years of age. Patient II.2 never achieved independent walking. Today, both patients are wheelchair-bound. Patient II.1 experienced a unique episode of prolonged hyperthermic at 10 months of life and received valproate until the age of 3 years with no recurrent episodes. Both presented pyramidal syndrome with hyperreflexia, Babinski sign, and spasticity. Excessive weight gain started at 18 months of life for patient II.1 and 6 months of life for patient II.2. At 7 years of age, the weight of patient II.1 is over +4 SD and over +5 SD for patient II.2 at 4 years of age while height remained on the normal range (+1 SD and +2 SD, respectively). Currently, the father has a body mass index (BMI) of 24 and the mother has a BMI of 31.5, with a gain of weight in motherhood. No dysmorphic features were observed except a short columella. The neck was short with acanthosis nigricans. Brain magnetic resonance imaging showed cerebral atrophy for patient II.1 (Fig.1C, left panel), and partial agenesis of the corpus callosum with a lipoma as well as delayed myelinization for patient II.2 (not shown). Multiple ENT infections motivated amygdalectomy for both patients. Endocrine and metabolic screening showed no abnormalities except a raised parathyroid hormone (PTH) (57 ng/L, *N* = 10–46) with normal calcemia (2.57 mmol/L, *N* = 2.2–2.7) in II.2 who was supplemented with vitamin D. Array-CGH (SpectralChip CC4-V0.3, Perkin Elmer, Waltham, Massachusetts, USA) showed no pathologic copy number variant (CNV).

**Table 1:** Clinical findings in patients with mutations in adaptor protein complex 4 (AP4) subunits

	II.1	II.2	Previously reported AP4-deficiency patients
Sex	M	F	Sex ratio ∼1
Age at evaluation (years)	7	4	From 2 to 24
ID	Severe	Severe	33/33
Speech	Less than 10 words, echolalia	Never acquired	30/31
Stereotypes	Hand flapping	Unmotivated laughter, hand flapping	26/28
Character	Calm	Episodes of heteroaggressivity	Shy, amicable, and calm for 14/19 unpublished (Abou Jamra et al. 2011; Verkerk et al. 2009) Shy and anxious for 2/19 (Abdollahpour et al. 2015)
Neonatal hypotonia	+	+	25/25
Pyramidal syndrome	+	+	33/33
Deambulation	Achieved then lost, wheelchair	Never achieved, wheelchair	31/31
Seizures	A unique episode of prolonged hyperthermic seizure at 10 months of life, treated with valproate until the age of 3 years without new convulsions	−	16/31
MRI examination	Cerebral atrophy	Incomplete corpus callosum agenesis and lipoma, delayed myelinisation	19/20 thin corpus callosum; ventriculomegaly; thinning and abnormal signal of the periventricular white matter; atrophy of the inferior vermis with cortical atrophy, dilated ventricles, prominent cisterns
Head circumference	−1.5 SD	−2 SD	Microcephaly for 25/32
Stature	+1 SD	+2 SD	Short stature for 12/14
Early onset of severe obesity	Starting at 18 months of life, weight >4 SD at 7 years	Starting at 6 months of life, weight >5 SD at 4 years	0/33

**Figure 1:**
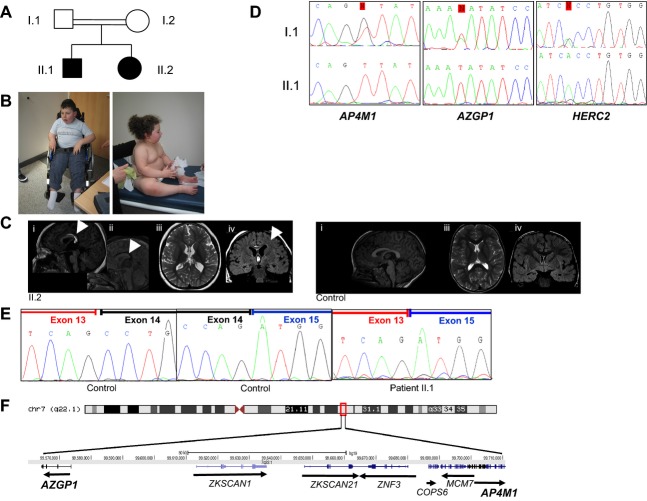
The combination of two substitutions in *AP4M1* and *AZGP1* genes underlies the association of two distinct syndromes in both patients. (A) Pedigree of the family. Shaded symbols indicate the affected individuals. (B) Photographs of patients II.1 (left) and II.2 (right). Legal representative of the patients gave consent for the publication of these photographs. (C) Brain MRI (magnetic resonance imaging) of patient II.2 aged 4 years old (left panel) and a 4-year-old normal control girl (right panel): sagittal T1 (i) and sagittal T1 fat sat (ii), axial T2-weighted Fast Spin Echo (FSE) images (iii) and coronal FLAIR-weighted FSE images (iv). Sagittal T1 (i) and sagittal T1 fat sat (ii) of the affected girl show a lipoma of the posterior part of the corpus callosum (white arrows). Axial T2 (iii) show a moderate ventricular dilatation compared to age-matched normal control. The coronal Fluid attenuation inversion recovery (FLAIR) (iv) of the affected girl shows delayed myelination of the white matter (arrow) compared to age-matched normal control. (D) Sequence analysis in a patient (II.1) and an healthy parent (I.1) showing the c.1137+1G>T; the c.595A>T, p.Asn199Tyr; and the c.12445G>A, p.Ala4149Thr variants in the *AP4M1*, *AZGP1*, and *HERC2* genes, respectively. (E) Exon 14 skipping in *AP4M1* mRNA from patients' fibroblasts compared to controls'. (F) Drawing showing the 7q22.1 region and the two mutated genes located 170 kb apart.

To identify the disease-causing mutation of this undiagnosed condition, we performed WES on peripheral blood DNA from all family members. We first focused on novel homozygous variants cosegregating with the disease and corresponding to either nonsynonymous (NS) variants, splice acceptor and donor site mutations (SS), or coding insertions/deletions (indels). We regarded variants as novel if they were absent from all publically available data sets, including those of dbSNP138 (http://www.ncbi.nih.gov/SNP), the 1000 Genomes Project (http://browser.1000genomes.org/index.html), the NHLBI ESP Exome Variant Server (http://evs.gs.washington.edu/EVS/), and from in-house exome data containing information for over 600 patient samples. Three candidate variants fulfilled these criteria (Table S1) and were confirmed by Sanger sequencing: a splice variant in the *AP4M1* gene (NM_004722.3: c.1137+1G>T), a misense variation in the *AZGP1* gene (NM_001185.3: c.595A>T, p.Asn199Tyr), and a misense variation in exon 81 of *HERC2* (NM_004667.4: c.12445G>A, p.Ala4149Thr) (Fig.1D). Filtering WES data for compound heterozygous mutations or heterozygous “de novo” mutations shared by the siblings did not provide any additional candidate variants.

The *AP4M1* splice variant affects the donor splice site of intron 14 (Fig.1D) and was described previously in five affected siblings from a consanguineous Moroccan family with a highly similar neurological presentation but no obesity was reported (Verkerk et al. 2009). As previously demonstrated by Verkerk and colleagues, this variant leads to skipping of exon 14 in the *AP4M1* transcript extracted from patients' cultured skin fibroblast (Fig.1E). *AP4M1* encodes one of the subunit (*μ*) AP4, an evolutionary conserved heterotetrameric complex consisting of two large (*α* or *β*), a medium (*μ*), and a small (*σ*) adaptin. Mutations affecting all four subunits of AP4 (AP4M1, AP4E1, AP4S1, and AP4B1) have been found to cause autosomal recessive AP4 deficiency syndrome characterized by severe ID, microcephaly, progressive spastic paraplegia, speech delay, and growth retardation (Table1 and Table S2) (Verkerk et al. 2009; Abou Jamra et al. 2011; Blumkin et al. 2011; Moreno-De-Luca et al. 2011; Najmabadi et al. 2011; Bauer et al. 2012; Philippe 2012; Kong et al. 2013; Tuysuz et al. 2014; Abdollahpour et al. 2015).

The p.Ala4149Thr substitution in *HERC2* affects an absolutely conserved amino acid residue located within the Regulator of Chromosome Condensation (RCC1) protein domain and is predicted to be damaging by various in silico tools (PolyPhen-2 score = 0.968, Sorting Intolerant from Tolerant (SIFT) score = 0, and disease causing according to MutationTaster, Charité University, Germany). The *HERC2* gene is located on 15q13.1 and encodes a large ubiquitin ligase protein. A distinct *HERC2* founder mutation (c.1781C>T, p.Pro594Leu) has been previously associated in the Amish community with a disorder characterized by mild developmental delay, autism spectrum disorder, and Angelman-like features (MIM:615516) (Puffenberger et al. 2012; Harlalka et al. 2013). Functional studies of the p.Pro594Leu variant demonstrated that it induces protein aggregation and decreased HERC2 abundance.

The third variant (c.595A>T, p.Asn199Tyr) affects the *AZGP1* gene, encoding the zinc-*α*2-glycoprotein (ZAG). The c.595A>T variant does not affect a highly conserved amino acid residue and its consequences on protein function is unclear (PolyPhen-2 score = 0.946, SIFT score = 0.01, and polymorphism according to MutationTaster). *AP4M1* and *AZGP1* are located on chromosome 7q22.1, 170 kb aside from each other (Fig.1F). To discriminate between a hot spot and a founder mutation, we performed genotyping analysis in all family members as well as in two affected siblings from the Moroccan family using microsatellite markers encompassing the *AP4M1* locus. As shown in Table S3, both families share a common haplotype supporting the hypothesis of a founder effect of the *AP4M1* mutation. The c.595A>T *AZGP1* mutation was not present in the previously described Moroccan family (Table S3). These data strongly support the hypothesis that the *AZGP1* mutation arose more recently in our family and occurs secondarily to the *AP4M1* mutation and on the same haplotype.

Our patients are severely retarded. They have some features of the autistic spectrum but marked and prolonged ocular contacts (Table1). Although two AP4 patients have been described with a shy and anxious character (Abdollahpour et al. 2015), the majority of patients with AP4 deficiency are described as shy, amicable, and calm (Table1) (Verkerk et al. 2009; Abou Jamra et al. 2011). Collectively, these data predicted only a minor effect of the p.Ala4149Thr *HERC2* variant identified in our family and demonstrated that the c.1137+1G>T *AP4M1* variant clearly accounts for the majority of neurological features observed in our patients.

However, none of the 33 previously reported AP4 deficiency cases are reported as obese, while obesity was described as a feature of the two siblings' condition as it started very early in both (before 1 year of age, table and sup clinical data). Similarly, no obesity has been reported in *HERC2*-mutated patients. While we cannot exclude that the *HERC2* variant may contribute to the neurological presentation of our cases, it is unlikely that it accounts for the obesity. By contrast, ZAG is an adipokine secreted by the adipose tissue and playing an important role in the mobilization and utilization of stored lipids (Balaz et al. 2014). Furthermore, several studies support the role of the ZAG protein in the regulation of body weight in both animal models and humans. Reduced plasmatic ZAG levels have been observed in ob/ob mice and *Azgp1* deficient mice showed increased body weight and decreased adipocytic lipolysis (Rolli et al. 2007; Mracek et al. 2010b). Moreover, oral administration of human ZAG to ob/ob mice resulted in progressive loss of body weight (Russell and Tisdale 2012). Linkage analyses showed linkage disequilibrium mapping of genes influencing human obesity, insulin resistance, and type 2 diabetes in the 7q22.1 region and the rs4215 SNP in *AZGP1* gene is associated with obesity in Chinese population (Sell et al. 1998; Li et al. 2003; Sim et al. 2011; Huang et al. 2012; Sanghera and Blackett 2012; Zhu et al. 2012). Finally, both lower serum concentrations of ZAG and significantly lower ZAG expression in the adipose tissue and liver have been observed in obese subjects (Selva et al. 2009; Mracek et al. 2010a; Garrido-Sanchez et al. 2012; Balaz et al. 2014). Further investigations will be needed to evaluate the ability of the mutant p.Asn199Tyr ZAG protein to induce lipolysis, but these results make *AZGP1* an excellent candidate gene for very early onset obesity in humans and may provide interesting clues for novel therapeutic interventions in obese patients.

Altogether, our results suggest that the phenotype observed in our patients results from the additional effects of *AP4M1* and *AZGP1* mutations accounting for the neurological signs on one hand and the precocious morbid obesity on the other hand. Additional subtle effects of the *HERC2* variant on the neurological presentation, particularly on the communicative skills, cannot be excluded. The current report further demonstrates how consanguinity could increase intrafamilial clustering of multiple hereditary diseases and how WES has considerably empowered our ability to detect such complex events.

The term contiguous gene syndrome (CGS) has been proposed in 1986 to explain the association of unrelated clinical features due to the deletion of multiple genes lying in close proximity to one another on a single chromosome (Schmickel 1986). The phenotypes observed arise as a result of the combination of the endophenotypes from each deleted gene sensitive to haploinsufficiency. While CGS is caused by a single mutational event (i.e., a chromosomal deletion), the phenotype we describe is the consequence of two independent mutational hits in two genetically linked genes. The mode of inheritance we describe is also different from a digenic inheritance that refers to the alteration of two interacting genes to cause a phenotype. While the final demonstration of our hypothesis is awaiting the identification of *AZPG1* mutations in obese patients with normal neurodevelopment as well as the demonstration of the impaired function of the mutant p.Asn199Tyr ZAG protein, we propose to use the name contiguous mutation syndrome to describe this complex and clinically challenging phenotype caused by independent mutations in genetically linked genes.

## Supporting Information

**Data S1.** Supplementary Materials and Methods **Table S1.** Number of variants identified by whole-exome sequencing. **Table S2.** Literature review of patients with mutation in one of the four AP4 genes. **Table S3.** Genotypes of the two families carrying the c.1137+1G>T mutation (Verkerk et al. 2009).
