# Supplementary material for: Contiguous mutation syndrome in the era of high-throughput sequencing
Source: Mol Genet Genomic Med. 2015 Mar 18;3(3):215–20. doi: 10.1002/mgg3.134 (PMC4444163; doi:10.1002/mgg3.134)
Supplement: Supplementary file 1 — Data S1. Supplementary Materials and Methods Table S1. Number of variants identified by whole-exome sequencing. Table S2. Literature review of patients with mutation in one of the four AP4 genes. Table S3. Genotypes of the two families carrying the c.1137+1G>T mutation (Verkerk et al. 2009). [file mgg30003-0215-sd1.docx]

**Supplementary Materials and Methods**

*Whole Exome Sequencing (WES)*

Agilent SureSelect librairies were prepared from 3 µg of genomic DNA sheared with a Covaris S2 Ultrasonicator as recommended by the manufacturer. Exome capture was performed with the 50 Mb SureSelect Human All Exon kit (Agilent technologies) using a multiplex approach with molecular barcodes for traceable ID of samples. Sequencing was carried with the SOLiD5500 (Life Technologies) on a pool of barcoded exome libraries. 75+35 paired-end reads were generated and mapped on human genome reference (NCBI build37/hg19 version) using LifeScope (Life Technologies).

Sequences produced allow respectable mean sequence coverage of 39-77 reads per bp position, with more than 74-85% of targeted bases covered at 15X. Sequence reads were aligned to the human reference genome sequence (assembly GRCh37) using Mapreads. SNPs/indels were called using Sequence Alignement/Map tools (SAMtools) Pileup. Poorly mapped (less than 3X cover) and low-quality reads (less than 20 quality score) were removed. An in-house software (PolyWeb) was used to annotate and filter the variants.

*Cell culture and RNA extraction*

Primary fibroblasts were cultured at 37°C under 5% CO2 in RPMI® + glutamax or OPTI-MEM® + glutamax supplemented with 10% of fetal bovine serum (FBS) and 5% of penicillin/streptomycin (complete medium) (Life Technologies). Total RNA was isolated using the RNeasy Mini Kit (Qiagen) according to manufacturer’s protocol. RNA concentration was measured by spectrophotometry (Nanodrop, Thermo Scientific).

**Supp. Table S1:** Number of variants identified by whole-exome sequencing

| **Patients** | **II.1** | **II.2** |
| --- | --- | --- |
| Total variants | 48036 | 52062 |
| Novel variants (dbSNP138/1000GP/EVS/in-house database) | 687 | 861 |
| Homozygous for patients and heterozygous for parents | 196 | 221 |
| Coding Non-synonymous/indel/consensus SS variants | 47 | 48 |
| Shared by both patients | 3 | 3 |

**Supp. Table** **S2**: Literature review of patients with mutation in one of the four AP4 genes

| **References** | ***AP4M1*** | ***AP4E1*** | ***AP4B1*** | ***AP4S1*** | **TOTAL** | **SEX** |
| --- | --- | --- | --- | --- | --- | --- |
| ([Verkerk et al., 2009](#_ENREF_9)) | 5/ c.1137+1G>T |  |  |  | 5 | 2F,3M |
| ([Moreno-De-Luca et al., 2011](#_ENREF_6)) |  | 2/192kb del |  |  | 2 | 1F,1M |
| ([Bauer et al., 2012](#_ENREF_3)) ([Blumkin et al., 2011](#_ENREF_4)) |  |  | 2/ c.664 delC |  | 2 | 1F,1M |
| ([Abou Jamra et al., 2011](#_ENREF_2)) |  | 2/c.542+1delGTAA | 3/ c.487_488insTAT | 3/c.124C>T | 8 | 4F,4M |
| (Philippe, thesis, 2012) |  |  | 4/ c.1159_1160delAC |  | 4 | 1F,3M |
| ([Najmabadi et al., 2011](#_ENREF_7)) | 1/c.577G>A | 1/c.1360_1361insAG |  |  | 2 | nd |
| ([Kong et al., 2013](#_ENREF_5)) |  | 2/ c.542+1delGTAA |  |  | 2 | 2F |
| ([Abdollahpour et al., 2014](#_ENREF_1)) |  |  | 2/ c.1160_1161delCA |  | 2 | 1F,1M |
| ([Tuysuz et al., 2014](#_ENREF_8)) | 2/ c.1012C>T ; 2/ c.952C>T |  | 2/ c.869delC |  | 6 | 5F,1M |
| This report | 2/ c.1137+1G>T |  |  |  | 2 | 1F,1M |
| **TOTAL (August 2014)** | **12** | **7** | **13** | **3** | **35** | **18F,15M, 2nd** |

Nd; no data

**Supp. Table S3:** Genotypes of the two families carrying the c.1137+1G>T mutation ([Verkerk et al., 2009](#_ENREF_9))

|  |  |  | This report | | | | ([Verkerk et al., 2009](#_ENREF_9)) | |  |
| --- | --- | --- | --- | --- | --- | --- | --- | --- | --- |
| **markers** | **genes** | **NCBI build37 position** | **I.1** | **I.2** | **II.1** | **II.2** | **87RD35** | **87RD38** | **unrelated control** |
| D7S2409 |  | 91056453 | 219/221 | 219 | 219/221 | 219/221 | 219 | 219/223 | 219 |
| D7S657 |  | 92566477 | 258/260 | 250/256 | 256/258 | 250/258 | 246 | 256/258 | 250/260 |
| **D7S479** |  | 96093138 | 121/**123** | 117/**123** | 117/**123** | **123** | **123** | **123** | 121/123 |
| **D7S651** |  | 98308219 | 165/**171** | 159/**171** | **171** | **171** | **171** | **171** | 165/167 |
|  | *AZGP1* | 99565796 | A/**T** | A/**T** | **T** | **T** | **A** | **A** | A |
|  | *AP4M1* | 99704138 | G/**T** | G/**T** | **T** | **T** | **T** | **T** | G |
| **D7S2480** |  | 100055770 | **203**/215 | **203**/219 | **203** | **203** | **203** | **203** | 217/219 |
| D7S515 |  | 101703900 | 152/156 | 152 | 152 | 152 | 154 | 154 | 158 |
| D7S2420 |  | 106890015 | 282/288 | 284/288 | 284/288 | 284/288 | 280 | 280 | 286/288 |
